# Prostaglandin E2 involvement in mammalian female fertility: ovulation, fertilization, embryo development and early implantation

**DOI:** 10.1186/s12958-018-0359-5

**Published:** 2018-05-01

**Authors:** Jean Damascene Niringiyumukiza, Hongcai Cai, Wenpei Xiang

**Affiliations:** Family Planning Research Institute/Center of Reproductive Medicine, Tongji Medical College, Huazhong University of Science and Technology, Wuhan, 430030 Hubei China

**Keywords:** Blastocyst, Cumulus, Chemokine, Extracellular matrix, Fertilization, Implantation, Prostaglandin E2

## Abstract

**Background:**

Infertility in mammalian females has been a challenge in reproductive medicine. The causes of female infertility include anovulation, ovulated oocyte defects, abnormal fertilization, and insufficient luteal support for embryo development, as well as early implantation. Ovulation induction, in vitro fertilization and luteal support regimens have been performed for decades to increase fertility rates. The identification of proteins and biochemical factors involved in female reproduction is essential to further increase female fertility rates. Evidence has shown that prostaglandins (PGs) might be involved in the female reproductive process, mainly ovulation, fertilization, and implantation. However, only a few studies on individual PGs in female reproduction have been done so far. This review aimed to identify the pivotal role of prostaglandin E2 (PGE2), a predominant PG, in female reproduction to improve fertility, specifically ovulation, fertilization, embryo development and early implantation.

**Results:**

Prostaglandin E2 (PGE2) was shown to play a relevant role in the ovulatory cascade, including meiotic maturation, cumulus expansion and follicle rupture, through inducing ovulatory genes, such as *Areg*, *Ereg*, *Has2* and Tnfaip6, as well as increasing intracellular cAMP levels. PGE2 reduces extracellular matrix viscosity and thereby optimizes the conditions for sperm penetration. PGE2 reduces the phagocytic activity of polymorphonuclear neutrophils (PMNs) against sperm. In the presence of PGE2, sperm function and binding capacity to oocytes are enhanced. PGE2 maintains luteal function for embryo development and early implantation. In addition, it induces chemokine expression for trophoblast apposition and adhesion to the decidua for implantation.

**Conclusion:**

It has been shown that PGE2 positively affects different stages of female fertility. Therefore, PGE2 should be taken into consideration when optimizing reproduction in infertile females. We suggest that in clinical practice, the administration of non-steroidal anti-inflammatory drugs, which are PGE2 synthesis inhibitors, should be reasonable and limited in infertile women. Additionally, assessments of PGE2 protein and receptor expression levels should be taken into consideration.

## Background

Many couples suffer from infertility, which is a big challenge not only to them but also to their family, endocrinology and infertility specialists, and embryologists. Different assisted reproductive techniques (ART) are used to enhance fertility rates. However, despite the constant development of sophisticated equipment and procedures, infertility treatment is still complicated and has a low success rate.

Previous studies have provided evidence that prostaglandins (PGs) may play a pivotal role in female reproduction, particularly in ovulation, implantation, and menstruation; however, the mechanisms involved remain elusive [1, 2]. The non-steroidal anti-inflammatory drugs (NSAIDs) aspirin and indomethacin, which inhibit cyclooxygenase (COX), were reported to have adverse effects on gonadotropin-releasing hormone release, ovulation, fertilization and luteolysis [3].

There are five types of PGs (also known as prostanoids): prostaglandin E2 (PGE2), prostaglandin D2 (PGD2), prostaglandin PGF2α (PGF2 Alfa), prostaglandin PGI2 (PGI2) and thromboxane (THA2). These are ubiquitously secreted, mostly from inflamed cells. PGs are synthesized from arachidonic acid by the key enzyme COX, also called prostaglandin synthase protein (PTGS) [4, 5]. Each PG has a specific influence and mechanism in the female reproductive system. However, it is possible that the different PGs also act in a synergistic way to fulfill their biological functions. Although the role of PGs in the female reproductive system has been already described, little is known about the individual PGs as separate functional entities in female reproduction.

**Fig. 1:**
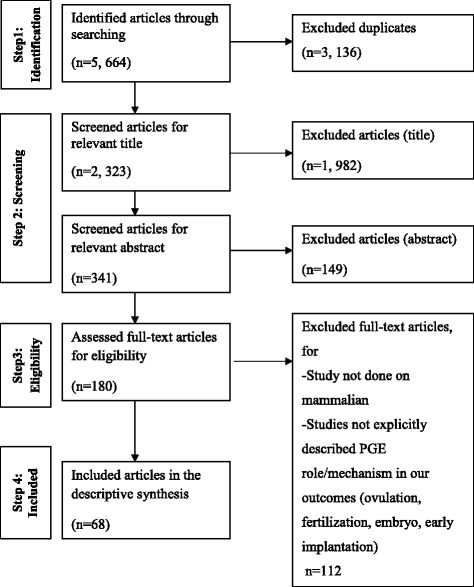
Selection of articles

PGE2 is the most common and substantial PG found in animal species. A wide range of receptor (EP, also noted as PTGER) subtypes (EP1, EP2, EP3 and EP4) that are bound by PGE2 have a multitude of signal transduction properties [6, 7]. The essential role of PGE2 production in female fertility has been suggested in previous studies [7, 8]. Experiments with PGE2 receptor type2 (EP2)-deficient mice showed disturbances in ovulation, fertilization, embryo development and implantation [9, 10]. Due to the predominance of PGE2 and its broad biological functions mediated by its different types of receptors, this review summarizes the role and mechanisms of PGE2 in ovulation, fertilization, embryo development and successful early implantation (Fig. 1).

## Results

### PGE2 involvement in the ovulation cascade

The luteinizing hormone (LH) surge was demonstrated to be responsible for oocyte cumulus expansion and meiotic maturation [11, 13]. However, the small amounts of LH receptors expressed in cumulus cells and in the oocyte [12] suggest that LH acts via other mediators. PGE2 is one of the mediators found to promote oocyte cumulus expansion and maturation [14]. Previous studies reported that inhibiting PGE2 production in follicles or genetically knocking out PGE2 and PGE2 receptor expression could prevent ovulation [7, 8]. However, the mechanisms of PGE2 in oocyte cumulus expansion and meiotic maturation remain elusive.

#### PGE2 enhances cumulus expansion

Ovulation requires adequate cumulus cell expansion to enable cumulus-oocyte complex (COC) detachment from the follicle wall and follicle rupture to release the oocyte into the oviduct. It was found that the LH surge increases PGE2 levels in the dominant follicle to modulate the action of gonadotropin for cumulus cell expansion and the expression of proteases associated with follicle rupture [15–18]. Female mice deficient in EP2, a PGE2 receptor, presented with abnormal cumulus cell expansion and unruptured follicles, which resulted in infertility or sub-fertility [7, 19].

The essential genes that are induced in COCs and are responsible for expansion include the following: oocyte maturation genes of epidermal growth factor (EGF)-like factors, such as amphiregulin (*Areg)*, epiregulin (*Ereg)* and betacellulin (*Btc)* [20, 21]; and matrix-forming and stabilizing elements, such as hyaluronan synthase 2 (*Has2*) and tumor necrosis factor α-induced protein 6 (*Tnfaip6*) [20, 22]. Studies have shown that PGE2 up-regulates the expression of many of these genes, including *Areg*, *Ereg*, *Has2* and *Tnfaip6,* in granulosa and cumulus cells via its receptor EP2 [21, 23]. Moreover, there is evidence that the cAMP pathway induces the expression of the cumulus expansion-related genes *Has2* and *Tnfaip6* in cumulus cells [4] and that PGE2 increases cAMP concentrations in cumulus cells during ovulation [23]; these findings suggest a direct role of PGE2 in cumulus expansion via these growth factors. The *r*ole of the protein kinase B and mitogen-activated protein kinases3/1 (PKB-MAPK3/1) pathway in cumulus expansion has also been documented [24, 25]*,* and *PGE2 was found to activate this pathway* for cumulus cell expansion and meiosis resumption [26].

#### PGE2 has been involved in oocyte meiotic maturation

PGE2 was found to be involved in not only cumulus expansion but also in meiotic maturation [27]. Cyclic adenosine monophosphate (cAMP) is a well-known mediator of meiotic maturation. PGE2 increases cAMP production in follicles, resulting in the maturation and cumulus expansion of oocytes [23, 28]. The PGE2 receptors EP2 and EP4, which are predominant in cumulus and granulosa cells [29], can increase intracellular cAMP levels when they are coupled to adenylate cyclase [30, 31]. In an in vitro study using mouse oocytes, treatment with an agonist selective for EP2 and EP4 increased cAMP production and subsequently increased ovulation rates [32], whereas the genetic manipulation of genes encoding EP2 and EP4 resulted in the inhibition of meiotic maturation and cumulus expansion [10, 33]. Several factors are responsible for maintaining spindle integrity during meiotic maturation. MAPK regulates spindle integrity during the meiotic maturation of oocytes [34, 35]. MAPK activity depends on phosphorylation. PGE2 was found to be responsible for the phosphorylation of MAPK [36], suggesting that PGE2 activates MAPK and indirectly induces the meiotic maturation of oocytes.

PGE2 was thought to mediate LH signals for meiotic maturation. Angiotensin II stimulation by LH has been reported to promote the meiotic maturation of oocytes by blocking the inhibitory effect of theca cells [37, 38]. It was demonstrated that the effects of angiotensin II in this process are mediated by PGE2 [39–41]. In an in vitro bovine oocyte study, indomethacin supplementation blocked the meiotic maturation of bovine oocytes induced by angiotensin II, whereas PGE2 treatment restored meiotic maturation to levels comparable to those induced by angiotensin II [39]. Human chorionic gonadotropin (hCG), a substitute for LH that stimulates oocyte maturation and ovulation in assisted reproduction, was reported to increase PGE2 and ovulatory gene expression through prostaglandin transport (PGT) in human granulosa cells [42].

**Fig. 2:**
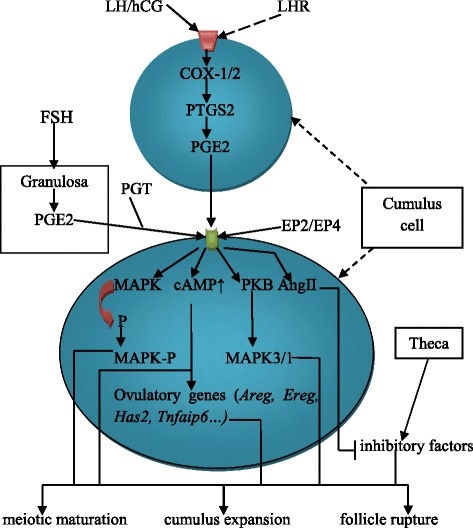
Ovulatory cascade. LH/hCG induces PGE2 synthesis and secretion in cumulus cells. Additionally, granulosa cells under FSH stimulation release PGE2, which is coupled with PGT and transported to the cumulus cell membrane to bind the PGE2 receptors EP2 and EP4. Via these receptors, PGE2 induces MAPK phosphorylation, increases intracellular cAMP levels and activates the PKB/MAPK3/1 pathway to stimulate ovulatory gene expression. Furthermore, PGE2 stimulates Angiotensin II to inhibit the inhibitory factors against meiotic maturation, released by theca cells

Even though LH and PGE2 were shown to trigger cumulus expansion and meiotic maturation separately [11–13], according to the above findings, we suggest similarity and synergetic effects between LH and the PGE2 pathways in regulating cumulus expansion and meiotic maturation. First, LH and PGE2 receptors are members of the G protein-coupled receptor (GPCR) family, and they trigger the ovulation process via activating adenylate cyclases to increase cAMP concentrations [23, 43, 44] for the synthesis of EGF-like factors, including *Areg* and *Ereg*, in granulosa and cumulus cells [23, 44–46]. Because the PGE2/cAMP pathway has been studied in meiotic maturation [23, 28], but not in the stimulation of *Areg* and *Ereg* secretion, we hypothesize that PGE2 induces cAMP pathways to stimulate EGF-like growth factors in cumulus and granulosa cells via its receptors (the same as LH); we propose this since these growth factors are positively involved in both cumulus expansion and oocyte meiotic maturation (Fig. 2).

### PGE2 modulates the fertilization process

#### PGE2 regulates cumulus ECM disassembly for sperm penetration into the oocyte

Cumulus cells synthesize and secrete extracellular matrices (ECMs) composed of mostly hyaluronan deposited in the intercellular space [47, 48]. ECM deposition in the inter-cumulus cell space leads to cumulus expansion, increases ECM viscosity and confers resistance capacity against biochemical and mechanical stress to the cumulus ECM. Chemokines were reported to modulate cumulus ECM consistency [49–51]. It has been demonstrated that upon ovulation, cumulus cells secrete various factors, including chemokine receptors and chemokines, such as CCL7, CCL2, and CCL9, which can increase the ECM viscosity to protect the oocyte from mechanical stress [49, 51]. The increased ECM consistency in cumulus cells by CCL-CCR signals impedes the passage of sperm and prevents fertilization [3, 49]. PGE2-induced activation of the cAMP pathway [50] disassembles and attenuates the ECM consistency [7, 50] by inhibiting the secretion of the chemokines CCL7 and CCL2 [50, 52]; these effects create a free space for sperm penetration.

**Fig. 3:**
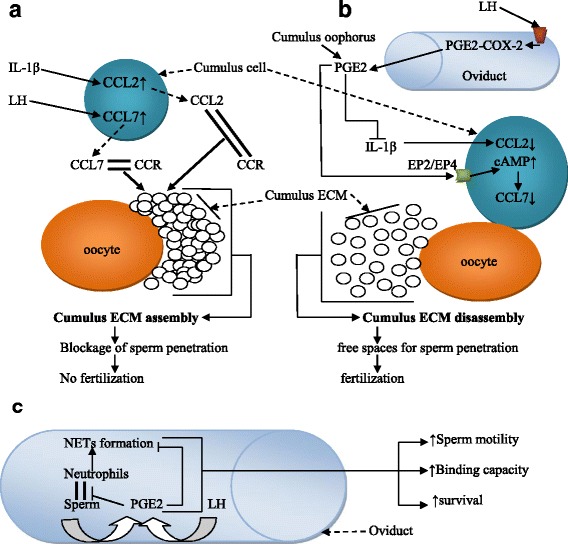
PGE2 in the fertilization process. PGE2 disassembles the cumulus ECM for sperm penetration and inhibits the phagocytic activity of PMNs against sperm. **a** Cumulus ECM assembly: In cumulus cells, LH and IL-1β respectively induce the expression of the chemokines CCL7 and CCL2. CCL7 and CCL2 bind the chemokine receptor CCR to induce cumulus cell-ECM assembly. **b** Cumulus cell-ECM disassembly: Upon ovulation, there is an increased level of PGE2 secreted by oviduct epithelial cells and the *cumulus oophorus*. PGE2 inhibits IL-1β and increases intracellular cAMP concentrations, respectively resulting in decreased expression levels of CCL2 and CCL7; these actions subsequently result in the disassembly of the cumulus ECM. The disassembly of the cumulus ECM leaves a free space for sperm penetration into the oocyte for fertilization. **c** In the oviduct, sperm binding to epithelial cells, as well as LH stimulation, induce PGE2 secretion. The released PGE2 inhibits neutrophil binding to the sperm and NET formation. Sperm in the presence of PGE2 have increased mobility, survival and binding capacity to the oocyte

In addition, interleukin-1β (IL-1β) stimulates the secretion of CCL2 in different cells [53, 54] and granulosa cells [55]. Given that cumulus cells are derived from granulosa cells, we hypothesize that IL-1β also stimulates CCL2 secretion in cumulus cells. It was found that PGE2 inhibits IL-1β in the myometrial cells of pregnant women [56], suggesting that PGE2 also inhibits the expression of CCL2 stimulated by IL-1β. Increased IL-1β expression was identified in cumulus cells lacking the PGE2 receptor EP2 [49], and we suspect a subsequent increase in CCL2 expression in these cells lacking PGE2 receptors. Taken together, we suggest that PGE2 acts via EP2 as a negative regulator of the chemokines CCL7 and CCL2 in the cumulus ECM to enhance ECM disassembly for sperm penetration and subsequent fertilization (Fig. 3a, b).

#### PGE2 protects sperm against phagocytosis in the oviduct

Neutrophils are mobilized in oviduct in response to the presence of sperm, a process resembling classical inflammation [57–59]. When protective signals become ineffective, the neutrophils fight against the sperms and reduce their motility and fertilization potential [60–62].

PGE2 exerts immunosuppressive activity [63], which inhibits the phagocytosis of sperm by neutrophils and macrophages [64, 65]. In the oviduct, in addition to the PGE2 secreted after epithelial cell stimulation by LH [66], it was found that sperm binding to the epithelial cells also induces PGE2 release [67], suggesting an auto-defense mechanism exerted by sperm in the oviduct. Via its receptor EP2, PGE2 may protect sperm from the phagocytic activity of polymorphonuclear neutrophils (PMNs) in the oviduct [68–70]. Neutrophils phagocytize spermatozoa either through cell attachment or by entrapping them in neutrophil extracellular traps (NETs) [71, 72]. A previous study demonstrated that PGE2 inhibits NET formation [73]. We suggest that the immunosuppressive potential of PGE2 and its inhibition of NET formation may result in enhanced sperm survival and higher motility and binding capacity of sperm in the oviduct, which subsequently increase the fertilization rate (Fig. 3c).

### Essential role of PGE2 in embryo development and early implantation

**Fig. 4:**
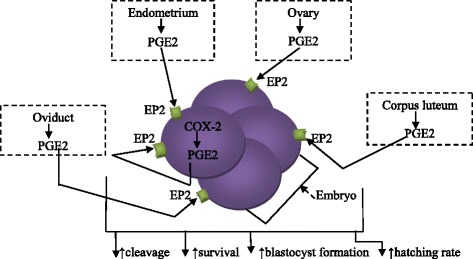
Role of PGE2 in embryo development. PGE2 is secreted by the embryo, from the 2-cell embryo to the blastocyst stage. The level of PGE2 increases gradually in the embryo pre-implantation. PGE2 secreted by the embryo, ovary, *corpus luteum*, oviduct and endometrium stimulate cleavage, survival, and blastocyst formation and hatching rates in the intrauterine cavity

Various factors mediate the cell proliferation, cleavage and survival of embryonic cells, as well as blastocyst formation and hatching. An embryo in the endometrium stimulates signals that have direct or indirect effects on its development and implantation. In embryos, gradually increasing levels of PGE2 from a 2-cell embryo to blastocyst were identified [74]. PGE2 was demonstrated to promote blastocyst hatching and enhance trophoblast proliferation after embryo transfer [75, 76]. PGE2 is considered to be a mitogenic, anti-apoptotic and angiogenic factor for cell proliferation and survival in other cells [77, 78], which suggests that PGE2 exerts these effects in embryo development, thus enhancing embryonic and trophoblast cell proliferation and survival for implantation (Fig. 4).

#### PGE2 maintains luteal function for early embryo development and implantation

After ovulation, mechanisms that protect the *corpus luteum* from regression should be initiated to stimulate the continuous production of factors protecting and promoting embryo development and early implantation. An increase in PGE2 biosynthesis and signaling in the uterus during the luteal phase was noted in sheep [79] and pig models [80], suggesting the role of this PG during early pregnancy. Through its receptor EP2, PGE2 induces the expression of LH receptors on the *corpus luteum*, resulting in an increase in progesterone (P4) synthesis [81, 82]. Previous studies reported that decreased blood flow to the luteal-containing ovary and decreased progesterone production result in luteolysis [83]. Blood flow has to be maintained for incoming bio-chemical factors, including LH, E2 and PGE2, to stimulate the *corpus luteum* to synthesize and secrete various factors, mostly P4, as well as to disseminate the secreted factors in the systemic system for embryo development and early implantation [84]. Via its receptor EP4, PGE2 was shown to enhance utero-ovarian blood circulation by increasing adenylate cyclase (AC) activity, which in turn increases nitric oxide synthase (OS) activity to increase synthesis and nitric oxide (NO) release, a vasodilator [85].

Interferon tau (IFN-t) was reported to reverse the PG secretion profile in endometrial cells during early pregnancy, thus resulting in increased PGE2 secretion and decreased PGF2a secretion [86, 87]. The increased PGE2/PGF2α ratio during early pregnancy is thought to prevent luteolysis and to support the luteal function for embryo development and successful implantation. It was reported that embryos secrete 17-β estradiol (E2) to stimulate continuous P4 production by the *corpus luteum* [88, 89]. The roles of embryo-secreted estradiol would be anti-luteolytic and lyteoprotectant, which are properties of PGE2. Previous studies demonstrated a simultaneous increase in E2 secretion by the embryo [90] and a shift from PGF2α to PGE2 secretion with a subsequent increase in the PGE2/PGF2α ratio during early pregnancy [91, 92]. Given the spatio-temporal pattern of E2 and PGE2 secretion and their localization, we hypothesize that there is an interaction between E2 and PGE2 to inhibit luteolysis. Moreover, E2 was shown to increase PGE2 production by stimulating PTGS2 secretion, which potentiates its activity through increasing the expression of EP2 [93]; E2 also decreases PGF2α production by inhibiting the secretion of PGFS (prostaglandin F2alpha synthase) and carbonyl reductase 1 (CBR1) [93], enzymes involved in PGF2α secretion [94–96].

**Fig. 5:**
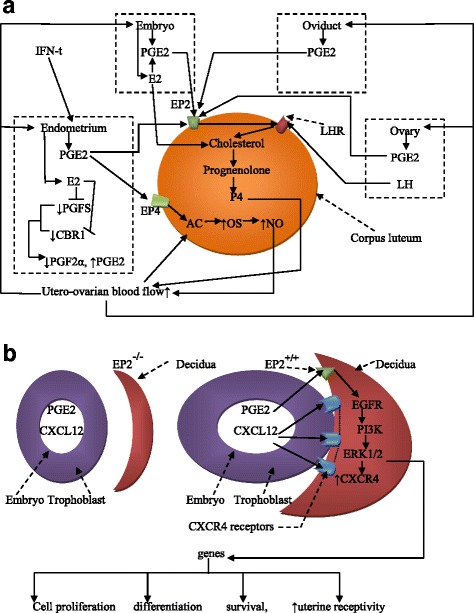
**a** PGE2 mediates early implantation. **a** Upon ovulation, PGE2 is secreted by the following reproductive organs: ovaries, endometrium and oviduct, as well as the embryo. EP2 induces LH receptor expression on the *corpus luteum* to secrete P4 and stimulate its continuous release into the in utero-ovarian circulation. P4 exerts positive feedback to the ovaries, endometrium and oviduct to recognize and maintain the ongoing implantation and embryo development. Via EP4, PGE2 increases the activity of adenylate cyclase (AC) and nitric oxide synthase (OS) successivelyto increase synthesis and nitric oxide (NO) release. NO, a vasodilator, increases the utero-ovarian blood flow and the concentration of pro-pregnancy factors. Additionally, E2 secreted by the embryo and endometrium stimulates PGE2/EP2 to increase the secretion of P4 and inhibits PGFS and CRB1, enzymes involved in the synthesis of PGF2α, an antagonist of PGE2. **b** In endometrial cells with EP2 receptors, PGE2 stimulates the expression of CXCR4 via the EGFR-PI3K/ERK1/2 pathway. CXCR4 is bound by the chemokine CXCL12, which is secreted by the embryo. PGE2 also stimulates the EGFR-PI3K/ERK1/2 pathway to induce the expression of genes involved in cell proliferation, differentiation and uterine receptivity

We hypothesize that PGE2 exerts its anti-luteolytic properties to protect and enhance embryo development and early implantation by inducing LH receptors on the *corpus luteum* for continuous progesterone secretion, by increasing nitric oxide (NO) synthesis and production to maintain adequate utero-ovarian blood flow and by mediating the effects of E2 and IFN-t to stimulate P4 secretion (Fig. 5a).

#### PGE2 stimulates chemokines for trophoblast apposition and adhesion to the maternal decidua

Different signals, including chemokines and chemokine receptors, produced in the endometrium are essential for embryo development and early implantation. Previous studies found that PGE2 modulates the expression of CXCR4, which is a receptor for the chemokine CXCL12, in hematopoietic stem cells [97] and stromal cells of the endometrium [98]. Embryonic signals were shown to regulate the expression of CXCR4 receptors in the endometrium [99], and the chemokine CXCL12 was identified in blastocysts [100].

PGE2 secreted by the embryo [101] binds to EP2 receptors in the endometrium to stimulate CXCR4 via the epidermal growth factor receptor (EGFR)-phosphatidyl inositol-3 kinase (PI3K) and extracellular signal-regulated kinase (ERK1/2) pathways [102]. The EGFR, PI3K and ERK1/2 pathways were shown to mediate early embryo implantation by inducing the expression of genes involved in cell growth, differentiation and uterine receptivity [103–105]. Moreover, high levels of CXCR4 expression were found in endometrium stromal cells at the apposition site of the embryo during the implantation window [106]. Furthermore, the PI3K/ERK1/2 pathways are known as pro-survival pathways that enhance the growth, proliferation, differentiation and survival of embryonic and endometrial cells [107]. We hypothesize that PGE2 secreted by embryos influences the apposition, adhesion and invasion of trophoblastic cells to the decidua by stimulating the expression of the chemokine CXCR4 receptor at the implantation site via the EP2, EGFR, PI3K and ERK1/2 pathways and/or by stimulating the synthesis of the chemokine CXCL12, which in turn will bind to CXCR4 receptors. In addition, we suggest that PGE2 uses the PI3K/ERK1/2 pathways to promote embryonic growth, proliferation, differentiation and survival, as well as endometrial cell growth, proliferation and differentiation in decidual cells.

The above findings indicate that PGE2 is essential for enhancing pre-implantation embryo development and early implantation. However, other mechanisms need to be further investigated (Fig. 5b).

## Conclusion

This review explored the involvement of PGE2 in cumulus cell expansion and meiotic maturation; in these processes, PGE2 either mediates LH signaling or directly acts on EGFs, such as Areg and Ereg, and matrix forming and stabilizing factors, such as *Has2* and *Tnfaip6.* PGE2 production in the oviduct reduces cumulus ECM viscosity around the oocyte for sperm penetration, protects the sperm from the phagocytic activity of neutrophils and enhances sperm survival, binding capacity, motility and function to promote successful fertilization. In addition, the effects of PGE2 on embryo development to a blastocyst, hatching and successfully implantation were demonstrated through the maintenance of luteal function and the induction and stimulation of chemokines for trophoblast proliferation, apposition and adhesion to the maternal decidua.

We suggest that in clinical practice, the administration of non-steroidal anti-inflammatory drugs, which are inhibitors of PGE2 synthesis, should be reasonable and limited in infertile women. Additionally, assessments of PGE2 protein and receptor expression levels should be taken into consideration in infertile women. However, we recommend more studies on the mechanisms of PGE2 in female reproduction and randomized clinical trials to draw definite conclusions to support this hypothesis.

## Abbreviations

AC: Adenylate cyclase
Areg: Amphiregulin
cAMP: Cyclic adenosine monophospate
CBR1: Carbonyl reductase 1
CCL2: Chemokine ligand 2
CCL7: Chemokine ligand 7
CCR: Chemokine receptor
COX: Cyclo-oxygenase
CXCL12: Chemokine 12
CXCR4: Chemokine receptor type 4
E2: Estradiol
ECM: Cumulus extracellular matrix
EGF: Epidermal growth factor
EGFR: Epidermal growth factor receptor
EP2: Prostaglandin E2 receptor type 2
EP4: Prostaglandin E2 receptor type 4
Ereg: Epiregulin
ERK1/2: Extracellular-regulated kinase 1and 2
FSH: Follicle stimulating hormone
Has2: Hyaluronan synthase 2
hCG: Human chorionic gonadotropin
IFN-t: Interferon-tau
IL-1β: Interleukin-1 beta
LH: Luteinizing hormone
MAPK3/1: Mitogen-activated protein kinases 3 and 1
NETs: Neutrophil extracellular traps
NO: Nitric oxide
OS: Oxide synthase
P4: Progesterone
PG: Prostaglandin
PGE2: Prostaglandin E2
PGF2α: Prostaglandin F2 alfa
PGFS: Prostaglandin F2 alfa synthase
PGT: Prostaglandin transport
PI3K: Phosphatidyl inositol-3 kinase
PKB: Protein kinase B
PTGS: Prostaglandin synthase
Tnaif6: Tumor necrosis factor α-induced protein 6
